# Effects of Chitosan and *N*-Succinyl Chitosan on Metabolic Disorders Caused by Oral Administration of Olanzapine in Mice

**DOI:** 10.3390/biomedicines12102358

**Published:** 2024-10-16

**Authors:** Balzhima Shagdarova, Viktoria Melnikova, Valentina Kostenko, Mariya Konovalova, Vsevolod Zhuikov, Valery Varlamov, Elena Svirshchevskaya

**Affiliations:** 1 Research Center of Biotechnology of the Russian Academy of Sciences, 119071 Moscow, Russia; vsevolod1905@yandex.ru (V.Z.); varlamov@biengi.ac.ru (V.V.); 2 Koltzov Institute of Developmental Biology of the Russian Academy of Sciences, 119334 Moscow, Russia; v_melnikova@mail.ru; 3 Shemyakin-Ovchinnikov Institute of Bioorganic Chemistry of the Russian Academy of Sciences, 117997 Moscow, Russia; biolentina.kost@mail.ru (V.K.); mariya.v.konovalova@gmail.com (M.K.); esvir@mail.ibch.ru (E.S.)

**Keywords:** chitosan, *N*-succinyl chitosan, olanzapine, orlistat, lipid metabolism, mice model, chemokines, PCR

## Abstract

Background: The issue of human mental health is gaining more and more attention nowadays. However, most mental disorders are treated with antipsychotic drugs that cause weight gain and metabolic disorders, which include olanzapine (OLZ). The search for and development of natural compounds for the prevention of obesity when taking antipsychotic drugs is an urgent task. The biopolymer chitosan (Chi) and its derivatives have lipid-lowering and anti-diabetic properties, which makes them potential therapeutic substances for use in the treatment of metabolic disorders. The purpose of this work was to analyze the effect of the natural biopolymer Chi, its derivative *N*-succinyl chitosan (SuChi), and Orlistat (ORL) as a control on the effects caused by the intake of OLZ in a mouse model. Methods: Mice were fed with pearl barley porridge mixed with OLZ or combinations OLZ + Chi, OLZ + SuChi, or OLZ + ORL for 2 months. The weight, lipid profile, blood chemokines, expression of genes associated with appetite regulation, and behavior of the mice were analyzed in dynamics. Results: For the first time, data were obtained on the effects of Chi and SuChi on metabolic changes during the co-administration of antipsychotics. Oral OLZ increased body weight, food and water intake, and glucose, triglyceride, and cholesterol levels in blood. ORL and SuChi better normalized lipid metabolism than Chi, decreasing triglyceride and cholesterol levels. OLZ decreased the production of all chemokines tested at the 4th week of treatment and increased CXCL1, CXCL13, and CCL22 chemokine levels at the 7th week. All of the supplements corrected the level of CXCL1, CXCL13, and CCL22 chemokines but did not recover suppressed chemokines. SuChi and ORL stimulated the expression of satiety associated proopiomelanocortin (POMC) and suppressed the appetite-stimulating Agouti-related protein (AgRP) genes. All supplements improved the locomotion of mice. Conclusions: Taken collectively, we found that SuChi more than Chi possessed an activity close to that of ORL, preventing metabolic disorders in mice fed with OLZ. As OLZ carries positive charge and SuChi is negatively charged, we hypothesized that SuChi’s protective effect can be explained by electrostatic interaction between OLZ byproducts and SuChi in the gastrointestinal tract.

## 1. Introduction

Nowadays, there is a progressive increase in the number of psychiatric disorders, the treatment of which often requires long-term antipsychotics. The increase in body weight and the development of obesity with the use of antipsychotics has been known since their discovery [1]. Olanzapine (OLZ) is an atypical antipsychotic drug (AAP) used primarily to treat schizophrenia and bipolar disorders. The main side effects are drowsiness and weight gain [2]. However, OLZ is still actively used today due to its effectiveness in the treatment of schizophrenia, as well as of anorexia nervosa and nausea caused by chemotherapy [3]. AAPs, in addition to weight gain, have side effects on the autonomic nervous and cardiovascular systems, as well as on the gastrointestinal (GI) tract. To reduce such effects, appropriate additional treatment is recommended along with AAP therapy. Plant extracts can act as such auxiliary substances [4,5,6].

A substance that also can mitigate the side effects of AAPs is biopolymer chitosan (Chi). The main mechanism of Chi action in the body is the reduction in the absorption of fat and cholesterol in the GI tract. Studies in obese mice have shown that Chi activates the leptin signaling pathway (JAK2-STAT3) and thus reduces tissue resistance to leptin and inhibits adipogenesis [7,8]. It has been shown that Chi reduces the level of triacylglycerol and cholesterol in the liver in mice receiving a high-fat diet (HFD). It has also been shown that Chi inhibits weight gain in obese animals and significantly reduces blood lipid levels in animal models of hyperlipidemia [9,10]. In the work of Chiu et al., the addition of Chi significantly reduced total cholesterol levels in the liver and increased cholesterol levels in feces in rats on HFD. The expression of the liver receptor-α protein activated by the peroxisomal proliferator (PPARα) in rats receiving the HFD was markedly reduced, which could be significantly changed with the help of Chi supplements. Chi drugs effectively reduced hypercholesterolemia and regulated cholesterol homeostasis by activating and inhibiting hepatic (AMPKα and PPARα) and intestinal (ACAT2) cholesterol modulators, respectively, as well as modulating downstream signaling pathways [11].

In addition, it is known that changes in the gut microbiota are also associated with weight gain and metabolic changes. The gut microbiome is associated with an adaptive immune response and inflammation, which is closely related to the pathogenesis of schizophrenia. The inclusion of pre/probiotics in the patient’s diet during the administration of AAPs helps to combat intestinal dysbiosis and prevent the progression of metabolic syndrome [12]. According to the literature data, Chi can have a beneficial effect on the intestinal microbiota [13] and thus can act as a protective substance when taking AAPs. Although multiple papers demonstrated anti-obesity effects of Chi both in mice and humans, the mechanisms of its activity is not well defined. We hypothesized that the major activity of Chi relates to its flocculant effect in the GI tract [14]. Currently, there are no data on the effect of Chi on AAP-induced metabolic changes.

Due to the presence of reactive groups, Chi can be subjected to chemical modifications while changing the chemical and biological properties of the polymer. Thus, the introduction of carboxyl groups into the Chi structure is one of the ways to increase its solubility, as well as to give it a negative charge. *N*-succinyl chitosan (SuChi) is the most inert nontoxic biopolymer for eukaryotic cell lines [15]. SuChi’s low toxicity results from its negative charge, which is compatible with mammalian cells. As OLZ carries a positive charge it can interact with SuChi in the GI tract. There are no data in the literature on the use of SuChi in obesity and metabolic disorders.

Orlistat (ORL) is an inhibitor of pancreatic and gastric lipases which covalently binds to the active center of lipases, thereby inactivating them. Due to the inhibition of gastrointestinal lipases, the triglyceride level in the bloodstream decreases. At the same time, an energy deficit is created, which leads to the mobilization of fat from the depot [16].

Taken collectively Chi, SuChi, and ORL have different mechanisms of activity. Which one can better affect weight gain and metabolic changes during the OLZ treatment of mice will be studied.

Chemokines are small blood proteins, produced mostly by epithelial and endothelial cells, which regulate leukocyte traffic through lymphoid and non-lymphoid tissues, cell survival, tissue regeneration, and homeostasis. Chemokines are classified into homeostatic and inflammatory chemokines [17]. The former are present in a high quantity in blood while inflammatory chemokines are produced mostly during inflammation. Cross-talk between tissues and the immune system is mediated by the expression of chemokine receptors on immune cells. Growing evidence suggests that chemokines play a role in schizophrenia [18,19]. The level of several chemokines (MCP-1, MIP-1α, IL-8, and IL-18) in peripheral lymphocyte cultures from first-episode schizophrenic subjects was significantly increased and some (RANTES) were decreased in a comparison with healthy controls [18]. AAPs (risperidone, olanzapine, and clozapine) partially corrected some chemokine production. The authors concluded that a dysregulated chemo-cytokine system is involved in the pathophysiology of schizophrenia. More recent studies found a genetic association of chemokine and chemokine receptor gene polymorphisms in schizophrenia [20]. Meta-analyses data showed an increase in CXCL8/IL-8, CCL2/MCP-1, CCL4/MIP-1β, and CCL11/eotaxin-1 in the blood of patients with schizophrenia [21]. A limited number of studies have analyzed the relationship between AAPs and the cytokine/chemokine axis [22,23,24]. Here we tried to analyze the effect of OLZ on chemokine level in mice when co-administered with chitosans.

Metabolic alterations associated with the AAP treatment of patients include insulin signaling, appetite dysregulation, altered hypothalamic energy sensing, and inflammation [25]. However, weight gain can also be induced by schizophrenia itself [26]. Grimm et al. suggested that obesity and weight gain are linked to the psychopathology of schizophrenia, namely with altered reward anticipation, which in turn is related to striatal dopaminergic dysregulation [27]. In our work, we estimated hypothalamic appetite associated gene expression based on the work of Singh et al., who demonstrated that OLZ significantly increased a panel of genes in the hypothalamus of mice treated with OLZ [28,29].

Finally AAPs can decrease anhedonic symptoms observed in schizophrenic and depressive patients, reverse cognitive disturbances, and increases neurogenesis [30]. In the present study, the effect of OLZ and compensatory effects of supplements (Chi and SuChi) on anxiety-related behavior and locomotor function was analyzed using an open field test.

The aim of this work was to analyze the effect of the natural biopolymer chitosan (Chi), its derivative *N*-succinyl chitosan (SuChi), and orlistat (ORL) as a control on the effects caused by the intake of olanzapine (OLZ) in a mouse model.

## 2. Materials and Methods

### 2.1. Materials

Chitosan with molecular weight (Mw) 1040 kDa and degree deacetylation (DD) 85% (Bioprogress LLC, Schelkovo, Moscow, Russia). Succinic anhydride and nitric acid (Sigma, St. Louis, MO, USA). Commercial preparations of olanzapine (OLZ) (5 mg tablets, Krka, d.d., Novo Mesto, Slovenia) and orlistat (ORL) (60 mg capsules, Polpharma SA, Starogard Gdański, Poland). Other reagents and solvents used in the study were of analytical grade.

### 2.2. Chitosan and Its Derivative

Low molecular weight chitosan (Chi) was prepared from high molecular weight chitosan by chemical depolymerization. For this purpose, high-molecular chitosan with an Mw of 1040 kDa and a DD of 85% was suspended in 6% nitric acid. The reaction was carried out at 70 °C with stirring for 6 h. After leaving it overnight, the precipitate obtained after hydrolysis was separated by filtration, resuspended in distilled water, and heated until its complete dissolution (70–80 °C). The solution was passed through a Schott filter to remove mechanical impurities. Next, the solution was cooled to 6 °C and the precipitate was filtered and then washed with isopropyl alcohol. The chitosan sample was additionally purified by precipitation from the water solution with the use of 12% ammonia. Then, the product was purified by dialysis in dialysis membranes (MWCO: 12,000–14,000) with multiple changes of distilled water. Finally, low molecular weight chitosan was lyophilized in a Martin Christ ALPHA 1-4LD plus freeze dryer (Christ, Osterode am Harz, Germany) using constant temperature (−50 °C) and vacuum pressure (0.04 mbar) for 20 h [31].

*N*-succinyl chitosan (SuChi) was obtained by the interaction of previously obtained low molecular weight Chi with succinic anhydride in dilute acetic acid according to the method in [32]. For this purpose, chitosan was dissolved in acetic acid, then a 4-fold excess of methanol was added, followed by the addition of succinic anhydride dissolved in a minimal volume of acetone under vigorous stirring (12 mole of anhydride/mole of chitosan amino groups). The reaction mixture was incubated without stirring for 16 h at 23 °C. Then the pH of the mixture was adjusted to 10 with sodium hydroxide solution and dialyzed with multiple changes of distilled water, then lyophilized in a Martin Christ ALPHA 1-4LD plus freeze dryer (Christ, Osterode am Harz, Germany) using constant temperature (−50 °C) and vacuum pressure (0.04 mbar) for 20 h.

The Mw of Chi was determined by high performance liquid chromatography using a GPC PSS NOVEMA Max analytical 1000 A column (PSS, Mainz, Germany). The control and analysis of chromatograms was carried out using the “Multikhrom” version 1.6 software (Ampersand LLC, Moscow, Russia). Pullulans (Mw = 342, 1260, 6600, 9900, 23,000, 48,800, 113,000, 200,000, 348,000, and 805,000 Da) (PSS, Mainz, Germany) were used as calibration standards.

^1^H-NMR spectra of Chi and SuChi were recorded on a Bruker AMX 400 spectrometer (Bruker, Billerica, MA, USA) operating at a ^1^H frequency of 400 MHz at 32 °C. Samples were prepared in deuterated water. 4,4-Dimethyl-4-silapentane-sulfonic acid was used as a standard. The solvent signal was suppressed by selective pulses using gradients.

### 2.3. In Vivo Experiments

Female mice of the C57BL/6 line at the age of 8–12 weeks were obtained from the Stolbovaya nursery (Moscow, Russia). The experimental protocol was approved by the Institutional Animal Care and Use Committee (IACUC) of the Animal Research Center of the Institute of Bioorganic Chemistry, the Russian Academy of Sciences (permission No. 327 dated 14 June 2021). Mice were kept in cages of 5 each without food and water restriction. To model metabolic changes, mice were caged 6 days a week for 7 weeks with 12 g of pearl porridge with peanut oil supplementation, in the groups of control (porridge only), OLZ, OLZ + Chi, OLZ + SuChi, and OLZ + ORL. ORL powder was taken out of gelatin capsules. OLZ was ground to powder in a mortar; the weights for the whole group were mixed with additives and porridge. The quantities of preparations are given in Table 1.

The weight of the animals was recorded weekly. The weight change was calculated from the initial and final weights of the mice. From the 3rd week onward, blood was drawn weekly and the levels of total cholesterol, triglycerides, and chemokines were determined. At weeks 4 and 7, mice were sedated with isoflurane and the hypothalamus was isolated to analyze the expression level of genes associated with possible metabolic disorders. Blood samples were collected from the retroorbital sinus under isoflurane anesthesia starting from the 3rd week of the experiment until the 7th week. Serum was separated after centrifugation at 3000 rpm for 10 min. The serum was stored in a freezer and used for further biochemical evaluations

### 2.4. Determination of Glucose, Triglycerides, Total Cholesterol, High-Density Lipoprotein, and Low-Density Lipoprotein in Blood Serum

For the quantitative determination of glucose, triglycerides, total cholesterol, low-density lipoprotein, and high-density lipoprotein in blood serum, we used kits with different methods produced by Olvex Diagnostikum LLC, Saint Petersburg, Russia.

For the quantitative determination of glucose content, we used a kit based on an enzymatic reaction under the action of glucose oxidase. For the quantitative determination of triglycerides in the blood serum of experimental mice, we used a kit based on a series of enzymatic reactions catalyzed by lipase, glycerokinase in the presence of ATP, glycerol-3-phosphate oxidase, and peroxidase. For the quantitative determination of total cholesterol in serum, a kit based on the enzymatic reaction under the action of cholesterol esterase was used. The color intensity of the reaction medium is proportional to the content of the studied parameter in serum and was determined using a Multiskan FC spectrophotometer (Termo Fisher Scientific, Waltham, MA, USA) at a wavelength of 490 nm.

A kit based on the ability of chylomicrons, very low density lipoproteins, and low density lipoproteins to precipitate in the presence of Mg^2+^ ions in interaction with phosphorus–tungstic acid was used for the quantitative determination of HDL in serum. After their precipitation, high-density lipoprotein cholesterol was determined in the supernatant by staining intensity at a wavelength of 490 nm. A kit based on the ability of low-density lipoproteins to precipitate at their isoelectric point at pH 5.10 in the presence of heparin was used to quantify LDL in serum. After centrifugation, the total concentrations of chylomicron cholesterol, very low density lipoprotein, and high density lipoprotein were determined in the supernatant. LDL concentration was calculated as the difference between the concentrations of total cholesterol and supernatant cholesterol. Concentrations were determined by the intensity of c staining at a wavelength of 490 nm.

### 2.5. Determination of Chemokine Levels in Blood

A multiplex chemokine assay was performed according to the manufacturer’s protocol. A standard 13-plex mouse chemokine panel (BioLegend, San Diego, CA, USA) was used. The analysis was performed using a FLEXMAP 3D cytometer (EMD Millipore, Billerica, MA, USA) and xPONENT software version 3.1 (EMD Millipore, Burlington, MA, USA). The mouse chemokines included in the kit were MCP-1 (CCL2), MIP-1a (CCL3), MIP-1b (CCL4), RANTES (CCL5), Eotaxin (CL11), TARC (CCL17), MIP-3a (CCL20), MDC (CCL22), KC (CXCL1), LIX (CXCL5), MIG (CXCL9), IP-10 (CXCL10), and BCL (CXCL13) MCP-1.

### 2.6. Hypothalamic Isolation

The hypothalamus was isolated in two mice at week 4 and three mice at week 7. Mice were sedated with isoflurane and opened; then, cardiac perfusion was performed to remove blood from organs, including the brain. After opening the cranium, the brain was removed and the hypothalamic area was excised with a scalpel on a dry Petri dish. The hypothalamic material was homogenized in 1 mL of ExtractRNA solution according to the manufacturer’s instructions (Eurogen CJSC, Moscow, Russia).

### 2.7. Quantitative Polymerase Reaction

Genomic DNA was removed using RNase-free DNAase according to the manufacturer’s instructions (Thermo Scientific, Waltham, MA, USA). The synthesis of cDNA from isolated mRNA was performed using a commercial reagent kit “Reverse transcriptase M-MuLV-RH” (BIOLABMICS LLC, Novosibirsk, Russia) containing MuLV revertase enzyme, deoxy nucleotide triphosphates, and enzyme buffer, using a random hexamer from the same kit according to the manufacturer’s protocol. The resulting cDNA was stored at −20 °C until the PCR was performed. Gene expression was analyzed by real-time polymerase chain reaction (qPCR)

For the real-time polymerase chain reaction, a commercial kit with BioMaster HS-qPCR SYBR Blue (2×) ready PCR mix (BIOLABMICS LLC, Novosibirsk, Russia) was used according to the manufacturer’s protocol. The reaction was performed in a volume of 20 μL using specific primers (Eurogen CJSC, Moscow, Russia). The list of primers is presented in Table 2.

### 2.8. Open Field Test

The testing apparatus consisted of a round box 40 cm in diameter and 40 cm in height. The platform was divided into sectors. Each animal was placed in the center of the apparatus, and behaviors were recorded for a period of 3 min in light and 1 min in dark conditions using a video tracking system. Locomotor activity was evaluated by measuring the total number of sector crossings and rearings.

### 2.9. Statistical Analysis

Graphs were created using MS Excel software version 14. Data are presented as the mean ± SEM (standard error of the mean) of two independent experiments; in each experiment, there were groups of 5 mice. Statistical analysis was performed using the Mann–Whitney U criterion. Significance levels of *p* < 0.05 were considered statistically significant.

## 3. Results and Discussion

### 3.1. Chitosan and N-Succinyl Chitosan

Chitosan is a unique polysaccharide that has great potential due to its polyelectrolyte properties as well as its high adsorption capacity, biodegradation, biocompatibility, low toxicity, lipid-lowering properties, and anti-diabetic properties [33,34]. However, the use of Chi with a high molecular weight is difficult due to the high viscosity of the solutions, and poor solubility in weak aqueous acid solutions. Chi can be fragmented in various ways, for example, by enzymatic hydrolysis [35]. However, this method is labor intensive and the use of highly purified enzymes is often not economically practical due to their high cost. Therefore, the use of acid hydrolysis is considered to be the most convenient, low-cost method, allowing a wide range of fragments of different molecular weights to be obtained by varying the reaction time and/or acid concentration. In the case of hydrolysis with nitric acid, the product also has the most attractive white color for the consumer, unlike chitosan obtained, for example, with hydrochloric acid. In this work, we used low molecular weight Chi with an Mw of 39 kDa, DD 90%, obtained by depolymerization using nitric acid.

**Figure 1:**
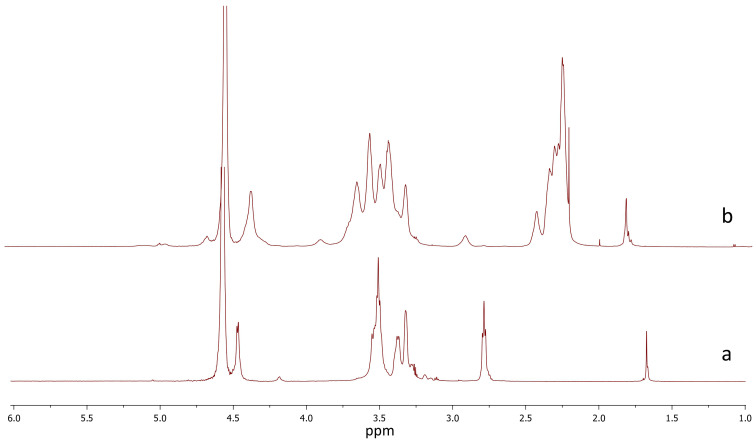
Stack of ^1^H NMR spectra of (**a**) chitosan and (**b**) *N*-succinyl chitosan.

Based on the obtained Chi, SuChi was synthesized using succinic anhydride. *N*-acylation is the most widely studied modification of Chi. However, the acid anhydrides used for the *N*-acylation of Chi are unstable and insoluble in aqueous medium. Therefore, the reaction is carried out under heterogeneous conditions, and methanol, acetic acid/methanol, etc. are used as solvents for the anhydrides. The degree of substitution depends on the ratio of reactants in the reaction, the time of the reaction, and the reaction temperature [36]. We carried out the reaction in an aqueous solution of acetic acid/methanol, with a 4-fold excess of methanol, since in this case the acylation proceeds predominantly on amino groups. An excess of 12 mole of anhydride/mole of chitosan amino group was used and the reaction was carried out for 16 h at 23 °C. We did not observe active jelly formation, possibly due to the use of a lower molecular weight chitosan sample. The degree of substitution was 70%. ^1^H NMR spectra of Chi and SuChi are presented in Figure 1. SuChi was water soluble and carried a negative charge, unlike chitosan.

It is known that the use of Chi with an MM of 48 kDa when feeding mice with high-fat food is effective and shows a lipid-lowering effect [8]. Also, Chi has been used in nanoparticle form only for OLZ delivery [37,38]. Neither Chi nor SuChi, however, have been used as substances to reduce metabolic changes induced by olanzapine administration.

### 3.2. Characterization of a Mouse Model of Oral Administration of Olanzapine

A high-fat diet is most commonly used as a model of obesity [39], but this model is not applicable when people start antipsychotics at normal weight. Previously, Cope et al. proposed an oral model to study the influence of AAPs in mice [40]. Female C57BL/6 mice were fed with high calorie peanut butter balls containing different AAPs (olanzapine, quetiapine, ziprasidone, and risperidone) and they gained weight [40]. In our experiments, peanut paste was replaced by pearl barley porridge with the addition of a small quantity of vegetable peanut oil, thereby decreasing the role of extra calories in the effects of OLZ.

**Figure 2:**
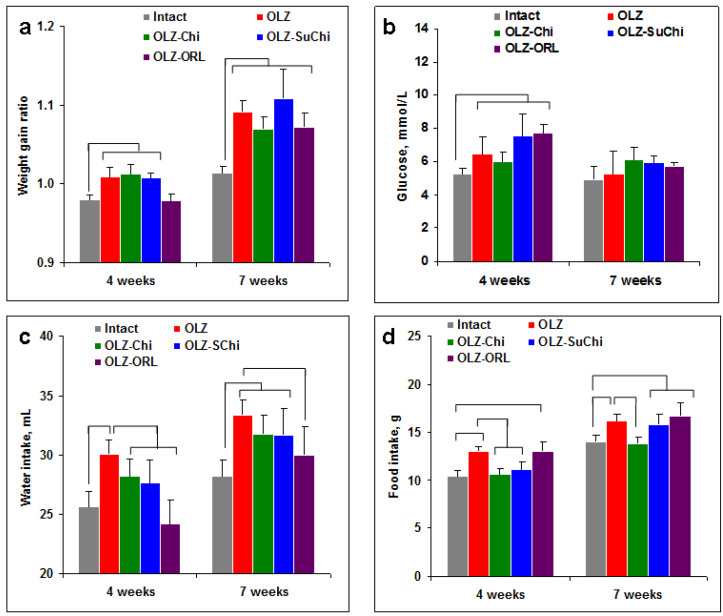
Effects of olanzapine (OLZ) and food supplements on body weight, glucose level, and water and food intake. Mice were fed with pearl barley porridge mixed with dry OLZ only or supplemented with chitosan (Chi), *N*-succinyl chitosan (SuChi), or orlistat (ORL). (**a**) Body weight was calculated as a ratio to the weight at the start of the experiment for each mouse and averaged. (**b**) Glucose level was estimated in the blood at the 4th and 7th weeks of the experiment. (**c**,**d**) Water (**c**) and food (**d**) intake were measured once a week from the 1st to 4th (4 weeks) and 5th to 8th weeks (7 weeks) per group of five mice and averaged. Data are shown as the mean ± SEM. Significant differences (*p* < 0.05, Mann–Whitney) are shown by brackets.

Oral OLZ administration resulted in a slight increase in the body weight starting from week 4 (Figure 2a). There was no significant effect of Chi and SuChi on the body weight while ORL partially compensated for the weight gain at the 4th week of the treatment. None of the supplements decreased the weight gain induced by OLZ (Figure 2a).

It is interesting that, at the 7th week, the glucose level in all OLZ groups significantly increased (Figure 2b). This effect was transient, possibly as an adaptation to OLZ, and was not found later. Weight gain in the OLZ group was associated with food and water intake (Figure 2c,d). ORL decreased water intake while only Chi decreased food intake.

### 3.3. Lipid Metabolism

**Figure 3:**
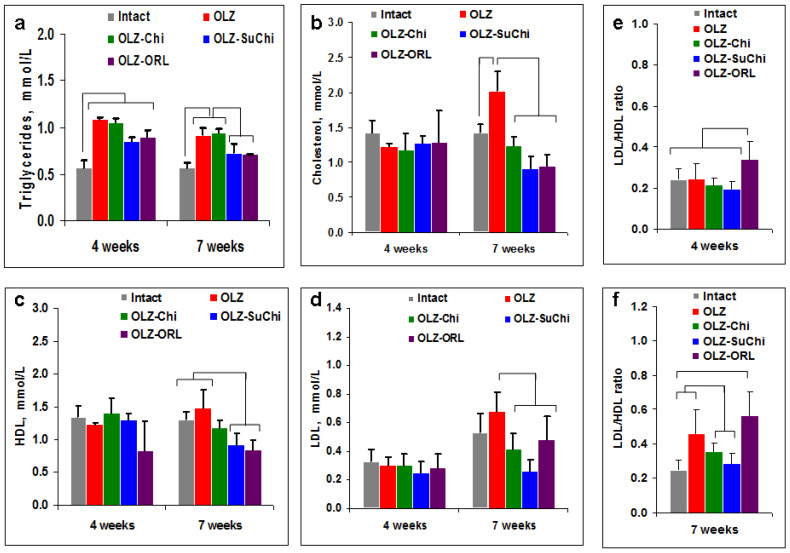
Effects of olanzapine (OLZ) and food supplements on lipid metabolism. Triglyceride (**a**), total cholesterol (**b**), high density lipoproteins (HDL) (**c**), low density lipoproteins (LDL) (**d**), and LDL to HDL ratios at the 4th (**e**) and 7th (**f**) weeks of the treatment in the blood of mice fed with dry OLZ only or supplemented with chitosan (Chi), *N*-succinyl chitosan (SuChi), or orlistat (ORL). Data are shown as mean ± SEM. Significant differences (*p* < 0.05, Mann–Whitney) are shown by brackets.

Weight gain is associated with an increase in triglyceride levels in the blood. Processes of triglyceride transport, storage (adipose tissue), repackaging (liver), and utilization (muscles) are strictly regulated [41]. By the 4th week of the experiment, triglyceride levels increased in all groups (Figure 3a). By the 7th week, SuChi and ORL, but not Chi, decreased the triglyceride concentration in the blood (Figure 3a).

Another parameter involved in many metabolic processes (hormone production, the synthesis of vitamin D and bile acids, and the construction of cell membranes) is blood cholesterol. When taking OLZ, as well as some other AAPs, an increase in cholesterol levels is often observed. We showed that on the 4th week of the experiment there were no differences in cholesterol levels between the groups (Figure 3b); however, on the 7th week, cholesterol was increased only in the OLZ group. Chi, SuChi, and ORL all prevented the increase in cholesterol. Earlier it was shown that ORL slightly reduces total-cholesterol, low and high density lipoproteins (LDL and HDL, accordingly), and triglyceride concentration [42]. Indeed, ORL also decreased HDL and LDL concentrations (Figure 3c,d). The anti-lipidomic effect of Chi and SuChi was comparable with that of ORL. However, the ratio of LDL to HDL was not favorable for ORL both at the 4th and 7th weeks (Figure 3e,f), while Chi and SuChi demonstrated a better effect.

### 3.4. Chemokine Regulation

**Figure 4:**
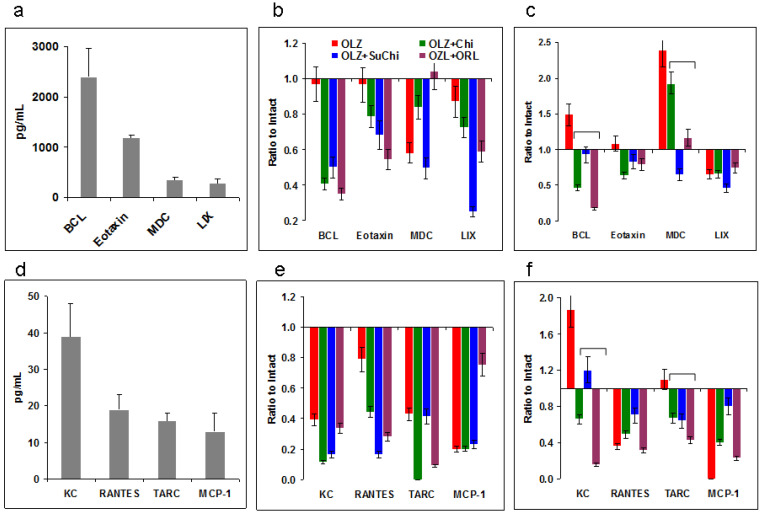
Effects of olanzapine (OLZ) and food supplements on blood chemokines. (**a**–**c**) Intact blood homeostatic chemokine concentrations (**a**) and the effect of OLZ and the combination of OLZ with chitosan (Chi), *N*-succinyl chitosan (SuChi), or orlistat (ORL) at week 4 (**b**) or 7 (**c**). (**d**–**f**) Intact blood inducible chemokine concentrations (**d**) and OLZ and OLZ plus the supplements at weeks 4 (**e**) and 7 (**f**) of the treatment. Data are shown and mean ± SEM (**a**) or as the ratios of experimental sample concentrations to the control intact blood ones. Significant differences (*p* < 0.05, Mann–Whitney) are shown by brackets only for the increased by OLZ chemokines.

Chemokines and their receptors are constitutively expressed by glial and neuronal cells of the central nervous system, where they participate in intercellular communication [17]. Chemokine signaling in the central nervous system plays a key homeostatic role, and chemokines are expressed in neurons, glia, and endothelial cells in bidirectional cross-interactions between parenchymal cells. Chemokine homeostasis also plays a role in obesity [43]. Information on the role of chemokines in schizophrenia and the effect of AAPs on their levels is limited. Here, we analyzed the level of 13 chemokines in the blood of mice treated orally with OLZ. Among them, the concentration of four chemokines (BCL, Eotaxin, MDC, and LIX) was higher than 100 pg/mL (Figure 4a) and these were found in all samples; thus, they are likely to be homeostatic. Another nine chemokines were of low concentration (0–70 pg/mL) and these are possibly inducible inflammatory chemokines. IP-10 was not found and two chemokines were found in the intact mice blood (MIG and MIP-1a) but not in most of the experimental samples. The latter (KC, TARC, RANTES, and MCP-1) were detected in almost all experimental samples.

The results in Figure 4 are shown as the ratios of blood concentration in the experimental samples to the intact ones. Evidently, OLZ suppressed chemokine production at the 4th week (Figure 4b,e) and stimulated BCL, MDC, and KC production at week 7 (Figure 4c,f).

Combinations of OLZ with food supplements decreased the chemokine levels even more, including those that were stimulated. Statistical differences are only shown for OLZ stimulated chemokines.

### 3.5. Expression of Appetite-Associated Genes in the Hypothalamus of Chitosan-Supplemented Olanzapine-Treated Mice

**Figure 5:**
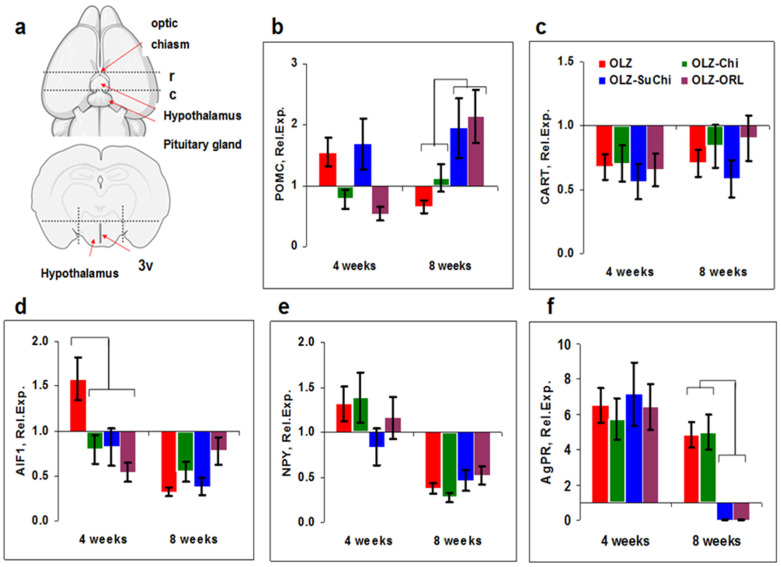
Expression of appetite-associated genes in the hypothalamus of olanzapine-treated mice. (**a**) Scheme of hypothalamic isolation. Incisions were made rostrally (r) at the level of the optic chiasma and caudally (c) along the pituitary pedicle, dorsally along the border of the third ventricle (3v). The drawing was created using the BioRender program (https://app.biorender.com). (**b**–**f**) An analysis of gene expression associated with metabolic changes in the hypothalamus of mice treated orally with olanzapine (OLZ) alone and in combination with orlistat (OLZ-ORL), chitosan (OLZ-Chi), or *N*-succinyl chitosan (OLZ-SuChi). Data are shown as the relative expression calculated as DeltaCt_experiment_/DeltaCt_control_, where experiment corresponds to OLZ-treated samples and control corresponds to intact mice. Significant differences (*p* < 0.05, Mann–Whitney) are shown by brackets.

For the analysis of gene expression, the hypothalamus of intact and experimental mice was isolated by incisions made rostrally (Figure 5a, (r)) at the level of the optic chiasma and caudally (c) along the pituitary pedicle, then dorsally along the border of the third ventricle (3v). Tissue was excised from each mouse and immediately lysed for RNA extraction.

Among nine proteins selected for analysis according to the literature [28], changes were observed in the expression of only five genes: *POMC*, *CART*, *AIF-1*, *NPY*, and *AgRP* (Figure 5b–f). The hypothalamus is the most important center for controlling food intake and energy homeostasis by changing the expression of specific genes. POMC is involved in various physiological processes, from pain relief to obesity. It is highly expressed in the hypothalamus, where peptides derived from POMC act as neuropeptides [44]. The stimulation of food intake by ghrelin in humans and rodents is explained, among other things, by a suppression of POMC expression [45]. We found that POMC expression was time dependent, and at week 4 it was slightly increased and then decreased to week 8 (Figure 5b). The effect of SuChi and ORL, but not Chi, was found only at the 8th week.

CART–peptide neurotransmitters are distributed in areas that play a role in the regulation of food intake [46]. A decreased CART expression can lead to hyperphagia and weight gain [47]. CART expression was decreased in all groups more at the 4th week but also at the 8th week (Figure 5c), which corresponds to the body weight gain found above.

Allograft inflammatory factor-1 (AIF-1) is involved in chronic inflammatory processes [48]. In obese women, the expression of AIF-1 is often increased [49]. Earlier OLZ was shown to increase AIF-1 expression [28]. Indeed, we registered increased AIF-1 expression at week 4 but not later, at week 8 (Figure 5d). All supplements decreased AIF-1 expression.

Agouti-related peptide (AgRP) is produced in the hypothalamus by the AgRP/NPY neurons. AgRP is co-expressed with NPY and acts to increase appetite and decrease energy expenditure. AgRP and NPY expression were increased in the OLZ group at week 4 (Figure 5e,f), but only AgRP was still increased at week 8 (Figure 5f). The only effect of SuChi and ORL on AgRP was found at week 8. These results were close to those obtained by Lin et al., who showed that, in mice on an HFD, the expression of NPY at week 1 of feeding was unchanged and decreased at week 8 [50].

### 3.6. Animal Behavior Analysis

**Figure 6:**
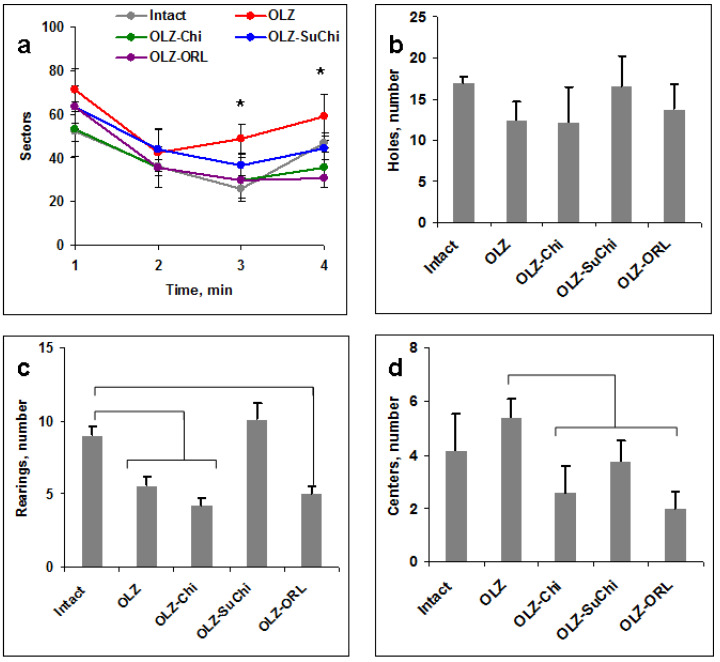
Effect of olanzapine (OLZ) and food supplementation on the locomotion and rearings of mice in an open field test (OFT). Mice fed with OLZ alone or supplemented with chitosan (Chi), *N*-succinyl chitosan (SuChi), or orlistat (ORL) were run for 4 min in an OFT (3 min in the light and the last min in the dark, shown by the gray bar). (**a**,**b**) Number of sectors crossed at the 4th (**a**) and 7th (**b**) weeks. (**c**,**d**) Total number of rearing at the 4th (**c**) and 7th (**d**) weeks. Significant differences (*p* < 0.05, Mann–Whitney) are shown by brackets (* in (**a**)).

The open field test (OFT) is widely used in the study of behavioral reactions in pharmacology and psychogenetics [51,52]. The OFT consists in the locomotion of mice in the round arena crossing the sectors and rearing at the walls or uprearing for 3 min in a bright light and for the last 4th min in a shaded light. The effect is estimated as the number of time-dependent sector crossings and total number of rearings. The test data are presented in Figure 6.

OLZ induced an increased number of sector crossings at the 3rd and 4th min in comparison with all other groups at 4 weeks of the treatment (Figure 6a), but significantly decreased the numbers of crossings at min 2 in comparison with Chi and especially SuChi groups at the 7th week of the testing (Figure 6b).

The total number of rearings demonstrated a decreased activity of OLZ-treated mice both at 4 and 7 weeks (Figure 6c,d). Only SuChi increased rearing activity at week 4 and all of the supplements increased it at week 7 (Figure 6c,d). ORL, at all times, kept locomotion close to the intact mice (Figure 6a,b).

## 4. Conclusions

A new oral murine model of OLZ-induced metabolic disorders with a limited quantity of oil consumption was developed. Three different molecules, all functioning in the GI tract, were studied as supplements to OLZ treatment. As the mechanism of OLZ activity is found in neurons and the GI tract, this permits differentiation between these two activities. We demonstrated that all supplements had small effects on body weight, which is likely to depend mostly on OLZ neuronal activity. However, all of them decrease blood lipids, with a better ratio of HDL to LDL demonstrated by Chi and SuChi than by OLR. So, the effect of polymers on lipid metabolism is likely to occur in the gut. Rather unexpectedly, we found that OLZ significantly decreased blood chemokine concentrations, both homeostatic and inflammatory. These data were not found earlier and possibly need studying in more detail. Among the 13 chemokines studied, a 7 week treatment with OLZ resulted in an increase in two homeostatic chemokines, BCL (CXCL13) and MDC (CCL22), and two inflammatory ones, KC (CXCL1) and TARC (CCL17), all of which were shown to take part in schizophrenia and other mental diseases. Among proteins affecting appetite we found only minimal effects of GI tract-associated supplements on gene expression. Namely, only the expression of the Agouti-related peptide (AgRP) gene was increased, six times, at week 4 of the treatment in comparison with intact mice, and was only partially corrected by SuChi and ORL at week 7. The only explanation for this interaction between the hypothalamus and the supplements unable to pass through the blood–brain barrier is the so-called gut–skin–brain axis functioning in many diseased conditions, in particular depression [53].

Taken together, we found that SuChi had a similar activity to ORL to a greater extent than Chi in preventing metabolic disorders in OLZ-fed mice. In the behavioral test, we observed a compensatory effect of SuChi, which brought the behavior of OLZ-fed mice closer to that of intact mice. Thus, our data show that Chi acts in the GI tract, and we believe that the metabolic syndrome is more caused by OLA byproducts during degradation in the liver and GI tract. Since OLZ is positively charged and SuChi is negatively charged, we hypothesized that the protective effect of SuChi could be explained by the electrostatic interaction between OLZ byproducts and SuChi in the GI tract.

## Figures and Tables

**Table 1:** Dosage of drugs and supplements (mg/kg·day).

Week	Olanzapine	Chitosan	*N*-Succinyl Chitosan	Orlistat	Peanut Oil, μL
1	6	300	300	50	40
2	8	400	400	100	40
3	10	500	500	200	40
4–7	12	600	600	300	40

**Table 2:** Primers used in the work [28].

№	Gene	Forward Primer	Reverse Primer
1	*NPY*	GTAACAAGCGAATGGGGCTG	TGATGTAGTGTCGCAGAGCG
2	*AgRP*	TGTGTAAGGCTGCACGAGTC	CATCCATTGGCTAGGTGCGA
3	*POMC*	CCATTAGGCTTGGAGCAGGT	GTGCGCGTTCTTGATGATGG
4	*CART*	TCCCTCTTTCCCCCAAAGGA	CACACCAACACCATTCGAGG
5	*TRPV1*	AGGGCCAGACAGCATTACAC	GGAACTTCACAATGGCCAGC
6	*TRPV3*	GCCAGGACCATCTTGGAGTT	CTTGTTTAAATCTGCTGTCCGTC
7	*GFAP*	GGCGAAGAAAACCGCATCAC	CTTGTGCTCCTGCTTCGAGT
8	*AIF-1*	TCCGAGGAGACGTTCAGCTA	CGTGTGACATCCACCTCCAA
9	*NF-kB1*	GAGGTCTCTGGGGGTACCAT	TTGCGGAAGGATGTCTCCAC
10	*GAPDH*	GGAGAGTGTTTCCTCGTCCC	ACTGTGCCGTTGAATTTGCC

## Data Availability

The original contributions presented in the study are included in the article, further inquiries can be directed to the corresponding author.
